# Brazilian Biomes as Promising Resources of *Rhodotorula* Yeasts for the Biotechnological Production of Carotenoids

**DOI:** 10.1002/cbdv.202500469

**Published:** 2025-06-16

**Authors:** David Cristian Rodrigues Lucas, Renan Campos Chisté

**Affiliations:** ^1^ Graduate Program of Biotechnology Institute of Biological Sciences, Federal University of Pará Belém Brazil; ^2^ Faculty of Pharmacy Universidade Federal de Minas Gerais (UFMG) Belo Horizonte Brazil

**Keywords:** Amazon, Atlantic Forest, Caatinga, Cerrado, Pampa, Pantanal

## Abstract

Yeasts belonging to the genus *Rhodotorula* are capable of synthesizing carotenoids, such as β‐carotene, γ‐carotene, torulene, torularodine, and astaxanthin. These carotenoids have been shown to offer health benefits to humans, such as immune system strengthening and a reduced risk of chronic degenerative diseases. This review systematically collected and analyzed extant literature on carotenoids of industrial interest produced by these yeasts found in Brazilian biomes (Pampa, Pantanal, Cerrado, Atlantic Forest, Caatinga, and Amazon). The most significant gaps are the absence of molecular identification of strains and the carotenoid composition. *Rhodotorula mucilaginosa* was found in all the biomes. The Cerrado biome had the largest number of *Rhodotorula* species, with seven species identified (*R. glutinis, R. mucilaginosa*, *R. graminis*, *R. aurantiaca*, *R. lactosa*, *R. toruloide*, and *R. diabovata*), followed by the Amazon biome, with four species: *R. mucilaginosa*, *R. minuta*, *R. aurantiaca*, and *R. glutinis*.

## Graphical abstract

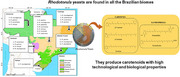

## 1 Introduction

Carotenoids are bioactive compounds synthesized in plants, microorganisms (bacteria, algae, filamentous fungi, and yeasts), but not by animals (except for hemipterans such as aphids, adelgids, phylloxerids), yet they can bioaccumulate them through food intake [1, 2]. Carotenoids are natural pigments, which can be mostly colored between yellow and red, and have biological properties that are greatly beneficial to human health, such as antioxidant, anti‐inflammatory, and anticancer properties, in addition to being some precursors of vitamin A. For these reasons, they have great value for the pharmaceutical, chemical, food, and feed industries [3, 4, 5].

Commercially, carotenoids are obtained from plants and chemical synthesis [6]. However, both production methods face some significant limitations. The availability of carotenoids derived from plant sources is subject to seasonal and geographical constraints, as these factors are inherently unpredictable and difficult to regulate. Moreover, chemical synthesis of carotenoids has been associated with the generation of hazardous waste, which is released into the environment.

The primary deleterious byproducts resulting from the synthesis of carotenoids through chemical means pertain to toxic organic solvents, including chloroform, dichloromethane, and dimethylformamide, among others. These solvents are often flammable and toxic, and whether improperly disposed of, they can cause atmospheric and water pollution. Reactive or unstable intermediates, such as organometallics (e.g., tin or lead compounds), halogenated agents, and alkylating compounds, pose a threat even in minute quantities. Furthermore, toxic by‐products, including polycyclic aromatic compounds (PACs), residual halogenates, and unstable free radicals, released during synthesis reactions, can possess mutagenic, carcinogenic, or bioaccumulative properties. Furthermore, strong acids and bases, such as sulfuric acid (H_2_SO_4_), hydrochloric acid (HCl), and sodium hydroxide (NaOH), employed for pH adjustment or catalysis, can result in soil and water contamination whether disposed of improperly [4, 7, 8].

The carotenoid market is very promising and will only grow over time. According to BCC Research [9], the global market for these molecules reached $2 billion in 2022, and is expected to reach $2.7 billion in 2027 with an annual growth rate of 5.7%. Among the known carotenoids, the main compounds sold globally are annatto, astaxanthin, β‐carotene, β‐apo‐8‐carotenal, β‐apo‐8‐carotenal‐ester, canthaxanthin, capsanthin, lutein, lycopene, and zeaxanthin [9]. For these reasons, investigations with microorganisms that produce carotenoids in Brazilian biomes have been conducted over time, and they have shown promising perspectives to provide high yields by sustainable means like the usage of low‐cost substrate as agro‐industrial wastes or natural substrates such as grape juice, grape must, peat extract and peat hydrolysate, sugar cane juice, and milk whey among others [4].

Brazil hosts one of the greatest biodiversity in our planet, as well as a large supply of microorganisms that produce carotenoids with useful properties for humans. In particular, yeasts of the genus *Rhodotorula*, the microorganism targeted by this review, can be found in all the Brazilian biomes: Amazon, Caatinga, Cerrado, Atlantic Forest, Pantanal, and Pampa [10, 11, 12, 13]. Among the Brazilian biomes, there are still few studies with *Rhodotorula* yeasts in the Amazon, given the vast forested plain that stretches between the Andes Mountains and the Atlantic Ocean over more than 5 million km^2^ in the north of the South American continent. Beyond the strict perimeter of the Amazon River watershed, the Amazon region extends to the Guiana coast to the north. Its climate is hot and humid with plenty of clouds and rain throughout the year [14]. This climate favors the development of a diverse microbiota, which is still largely unknown [15]. So far, six studies have reported about in this region, in the Pará State [16, 17, 18], Amazonas State [19, 20] and Roraima State Vital [18].

Therefore, in this review we collected information from Web of Science, Scopus, Pubmed, and Scielo databases using the following keywords: “yeasts”; “carotenoid production”; “carotenoid market”; “Brazilian biomes”; “carotenoid biosynthesis”; “carotenogenesis”; “Amazon”; “Amazonia”. “Cerrado”; “Caating”; “Atlantic Forest”; “Pantanal”; and “Pampa”. The papers from these databases were selected according to their relevance and the aim of this current paper. Then, this review was designed to gather systematic information about wide aspects related to carotenoid useful for interested industries, which are produce by yeasts from genus *Rhodotorula* and found in the Brazilian biomes, highlighting the Amazon biome.

## 2 Strengthening of the Immune System by Carotenoids

Carotenoids, including but not limited to β‐carotene, lycopene, lutein, and zeaxanthin, in conjunction with their inherent antioxidant properties, have been demonstrated to play a pivotal role in neutralizing the free radicals present within the human body, thereby reducing oxidative stress [21, 22].

Vitamin A is derived from carotenoids and plays a crucial role in maintaining the integrity of the skin and mucous membranes, which act as a barrier against pathogens [23]. Vitamin A has also been demonstrated to support the function of T and B cells in the immune system, thereby increasing the body's ability to combat infections [24].

The modulation of immune cells by carotenoids is achieved through the regulation of cytokine production, which are signaling molecules that mediate and regulate immunity. Carotenoids have been demonstrated to facilitate the activity of natural killer (NK) cells and macrophages, which play a pivotal role in the identification and eradication of infected or abnormal cells [25]. Certain carotenoids, including lycopene and astaxanthin, have been shown to possess anti‐inflammatory properties. These compounds have been demonstrated to play a crucial role in maintaining the balance of the immune system and preventing overreactions, a particularly salient feature in conditions such as autoimmune diseases [26, 27, 28].

## 3 Yeasts of Genus *Rhodorotula*

**FIGURE 1:**
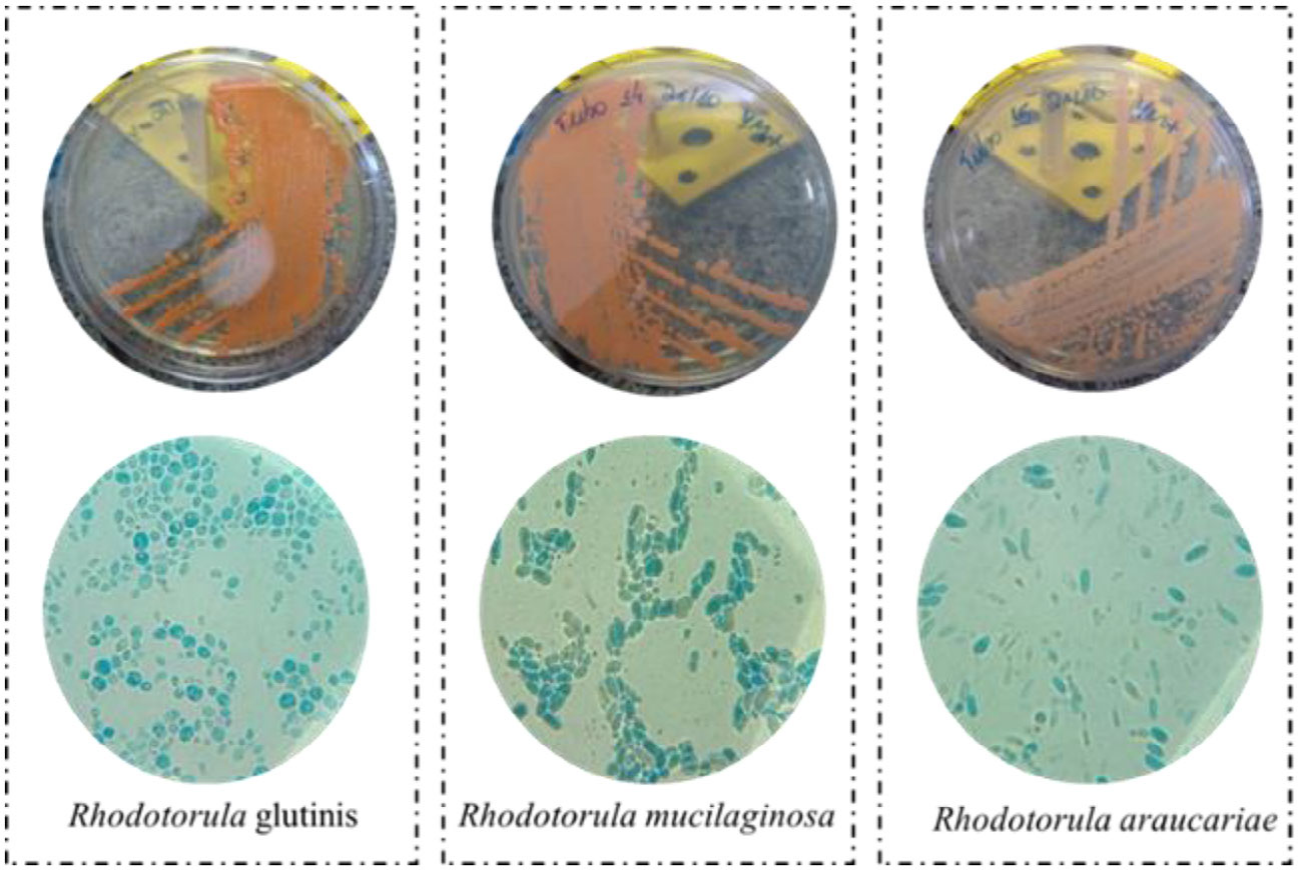
*Rhodotorula* yeasts and their subglobose, ovoid, ellipsoidal, and elongate forms. View on Petri plates and microscopy. Photo by Gilson Celso Albuquerque Chagas Junior.

*Rhodotorula* is a genus of unicellular pigment yeast, belonging to the phylum Basidiomycota, the family Cryptococcaceae, and the subfamily Rhodotorulodae. The first *Rhodotorula* found was in the 1930s in a cheese by Canadian microbiologist Charles Harrison [29]. The name of the genus comes from the words rhodos (which in Greek means red) and torula (which in Latin means protuberance). There are more than 164 species including *R. glutinis*, *R. toruloides*, *R. mucilaginosa*, *R. graminis*, among others. The cells of *Rhodotorula* yeast strains are polyphyletic (descended from two or more independent ancestors), appearing subglobose, ovoid, ellipsoidal, and elongated [30, 31, 32, 33], as illustrated in Figure 1.

The reproduction of *Rhodotorula* yeasts is asexual, normally carried out via multilateral and polar budding; on the other hand, it can also be sexual in some strains, occurring occasionally in the form of pseudohyphae [32]. These yeasts are ubiquitous saprophytes, as they feed on decomposing organic matter and are found in all spaces, practically [34]. The strains are capable of developing in different ecological and substrate conditions, even in environments with few nutrients. Approximately 50% of the population can be found in saltwater and freshwater [33, 35].

The most striking characteristic of these yeasts is, precisely, the biosynthesis of natural carotenoids, including their biomass, which is also of high quality and is a source of protein for the application as feed additives [33].

## 4 Main Carotenoids Produced by Yeasts From Genus *Rhodotorula*

More than 750 carotenoids were cataloged in the literature [36]. Most of them have an orange, reddish and yellowish color, but there are colorless structures, such as phytoene and phytofluene [2, 37]. The chemical structure of a carotenoid includes mostly isoprenoids of 40‐carbons formed by 8 units of isoprene with an extensive system of conjugated double bonds that generate resonance systems of electrons (π) moving throughout the polyene chain. Due to these structural characteristics, carotenoids are highly reactive and absorb electromagnetic radiation in the visible region (360–780 nm) [2, 38].

**FIGURE 2:**
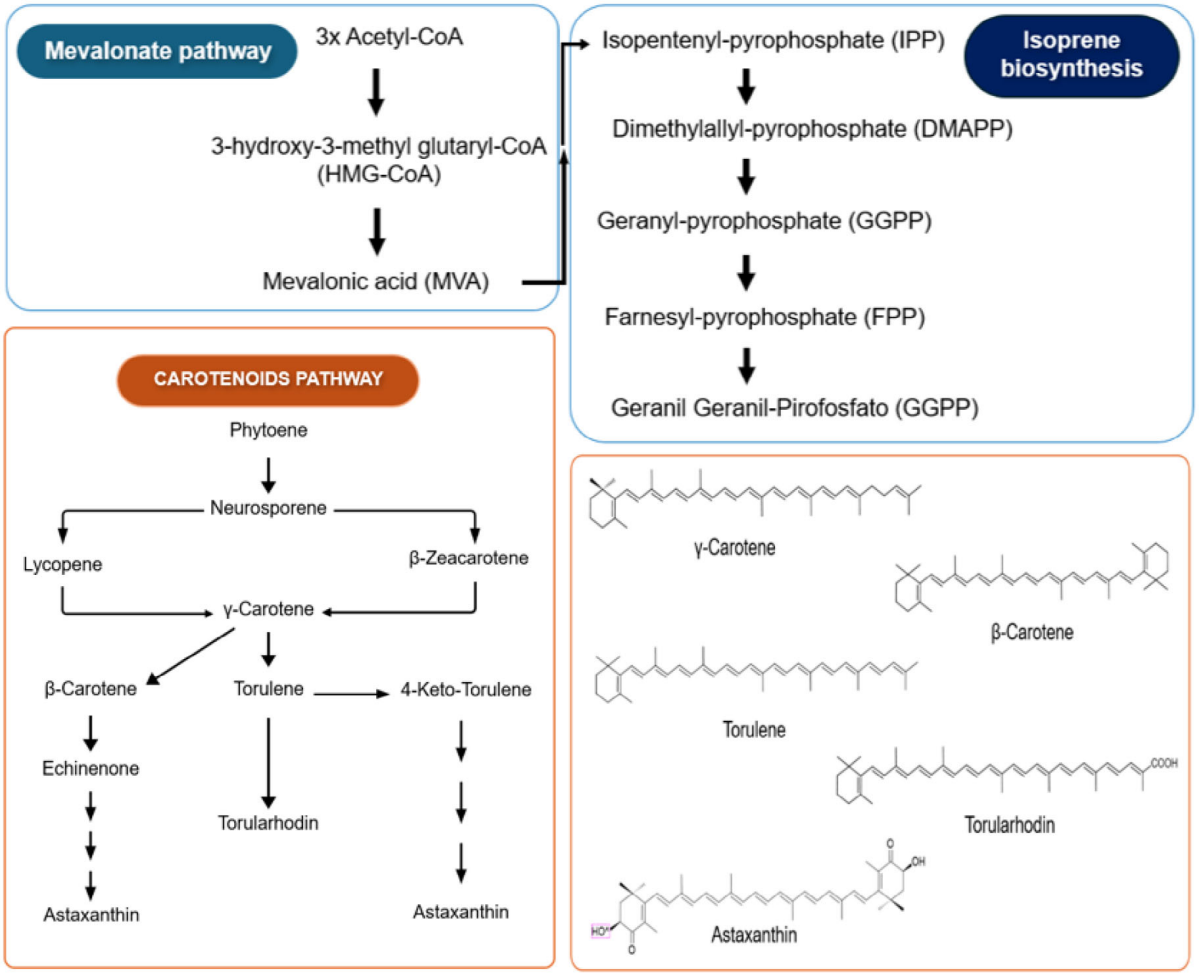
Biosynthetic pathway of the carotenoid production in yeasts.

Carotenoid biosynthesis in yeast involves three general steps, as shown in Figure 2. Briefly, (1) Conversion of Acetyl‐CoA into 3‐hydroxy‐3‐methyl glutaryl‐CoA (HMG‐CoA), catalyzing HMG‐CoA by HMG‐CoA synthase. Then HMG‐CoA is converted into the first precursor of the terpenoid biosynthetic pathway, mevalonic acid (MVA). MVA is phosphorylated by MVA kinase and then undergoes decarboxylation to become isopentenyl pyrophosphate (IPP). (2) IPP isomerized into dimethylyl pyrophosphate (DMAPP) with the addition of three IPP molecules, after which it is catalyzed by prenyl transferase into geranyl geranyl pyrophosphate (GGPP). By condensation of two GGPP molecules, phytoene (the first 40 carbons of the pathway) is produced, which undergoes denaturation to form lycopene. (3) Several cyclic carotenoids are derived from lycopene, such as β‐carotene, γ‐carotene, torulene, torularodine and astaxanthin when it undergoes various specific enzymatic reactions. Because lycopene is a *trans* compound, the isomerization of its first or second double bond occurs at the same stage as the denaturation reaction. Thus, γ‐carotene appears as a critical point in the branching because it acts as a precursor to β‐carotene and torulene in the carotenoid pathway in yeast. In turn, the hydroxylation and oxidation of torulene leads to the formation of torularhodin [2, 4, 39–44].

**FIGURE 3:**
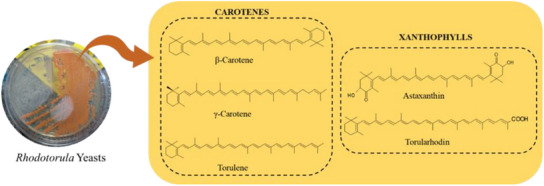
The main carotenoids reported for yeasts from genus *Rhodotorula*.

To stimulate the increase of the yield of carotenoids in *Rhodotorula* yeasts, optimization procedures on cultivation conditions have been investigated in both synthetic and alternative media, highlighting the use of agroindustrial waste as substrate to accomplish sustainable approaches [2, 45, 46]. Mutagenesis is referred as a conventional way to modulate carotenoid yield and composition in wild strains, including induction by UV radiation, high hydrostatic pressure, γ‐irradiation, *N*‐methyl‐*N*′‐nitro‐*N*‐nitrosoguanidine (NTG) and ethyl‐methane sulfonate (EMS), but engineering and metabolic engineering manipulations using *Escherichia coli* are the most recent approaches to produce the exogenous carotenoids [33]. The chemical structures of carotenoids can change through the action of chemical reactions such as hydrogenation, dehydrogenation, cyclization, double bond migration, chain shortening or extension, rearrangement, isomerization, introduction of substituents, and oxidation [47, 48, 49, 50]. Depending on the chemical structure, carotenoids can be classified into two large groups: carotenes, which are formed by hydrocarbons in their chemical structure, and xanthophylls, which are oxygenated derivatives of carotenes and may have different functional groups such as hydroxyl, ketone, carboxylic acid and epoxy [1, 2]. The most frequent carotenoids reported in *Rhodotorula* yeasts are β‐carotene, γ‐carotene, torulene, torularhodin, and astaxanthin (Figure 3).

β‐Carotene (Figure 3) is one of the well‐known pigments in nature and widely used by industries focused on food and feed additives, but also in pharmaceutical and cosmetics, among others. After ingestion, and depending on several individual and nutritional conditions, β‐carotene may be converted into two molecules of retinol (vitamin A) due to its two β‐ionone rings, and at least 11 carbons in the polyene side chain [2, 33]. Several fungi are capable of producing β‐carotene, namely *Blakeslea trispora* [51], *Phaffia rhodozyma* [52], *Rhodosporidium toruloides* [53], *Sporidiobolus salmonicolor* [43], and *Rhodotorula* spp. [54]. In relation to genus *Rhodotorula*, 70% of the carotenoids in them are made up of β‐carotene [55]. The frequent consumption of foods containing β‐carotene in their composition has been associated with a reduction in the development of chronic degenerative diseases, such as cardiovascular diseases and cancer, since carotenoids contribute mainly with antioxidant effects to inhibit or delay oxidative stress and damage [56, 57, 58, 59, 60]. Studies have also shown that increased intake of β‐carotene may reduce the risk of Parkinson's disease, especially in women [61, 62].

γ‐Carotene is formed after the enzymatic cyclization of lycopene (Figure 2) and considering that its structure has 11 conjugated double bonds and only 1 unsubstituted β‐ionone ring (Figure 3), this carotenoid has potential to form only one retinol molecule. This carotenoid is not often found in plants, but was reported in Amazonian fruits from palm species, such as buriti, peach palm, and tucumã [38, 63, 64]. Both the antioxidant properties and its biotechnological production have been reported for *Rhodotorula* and *Sporobolomyces* yeasts [65, 66].

Derived from the subtraction of two hydrogens from γ‐carotene, torulene is composed of 40 carbons and an unsubstituted β‐ionone ring linked to an 11‐carbon polyene side chain, giving 13 conjugated double bonds to the structure [67] (Figure 3). Theoretically, considering this structural requirement, one retinol molecule can be physiologically synthesized after ingestion of torulene. Among the yeasts of the genus *Rhodotorula*, *R. mucilaginosa* was reported to produce torulene in greater amounts (21.45 mg/L) than other carotenoids [41]. Due to the presence of a high number of conjugated double bonds in the chain, torulene was expected to show higher antioxidant activity than β‐ and γ‐carotene, which the protective effect against damages from oxidative stress in human prostate stromal cells was confirmed. Furthermore, the antimicrobial property of torulene was also previously reported [41, 68].

Derived from torulene, torularhodin has 40 carbons, a β‐ionone ring and a carboxylic acid group in its structure [44] (Figure 3). As observed for torulene, torularhodin shows high antioxidant activity due to the presence of a high number of conjugated double bonds, being reported as more efficient than β‐carotene for the scavenging capacity of reactive oxygen species, such as hydrogen peroxide (H_2_O_2_) and singlet oxygen (^1^O_2_) [69]. Moreover, according to Moliné et al. [70], torularhodin presented photoprotection mechanisms against UV‐B rays. In general, the same biological activities reported for torulene are expected to be extrapolated to torularhodin, yet these inferences are gaps in the literature to overcome by future investigations.

Finally, astaxanthin has 40 carbons, a long polyene chain with 13 conjugated double bonds, and since both the β‐ionone rings have hydroxyl and ketone groups (Figure 3), astaxanthin has no provitamin A activity. This carotenoid is the mainly responsible for the color of fishes such as salmon, trout, some crustaceans, and feathers of birds [71]. Astaxanthin is the second most commercially relevant carotenoid, which accounts for 26% of carotenoid sales worldwide, reaching $426.2 million in 2022 and is expected to reach $629.1 million in 2027 on the global market, with an annual growth rate of 8.1% for the period between 2022 and 2027 [9].

## 5 Carotenoid Market

In 2017, the global carotenoid market reached around $1.5 billion, and the latest statistics for 2022 showed the market's upward trend, around $2 billion and expected to reach $2.7 billion in 2027 with an annual growth rate of 5.7% [9]. Table 1 shows the values ($) and exported and imported quantities of carotenoids and their derivatives. The standard nomenclature of MERCOSUL (NCM) was used, which are NCM 32041810 and NCM 32041811 (carotenoids), NCM 32041912 (preparations containing β‐carotene, methyl or ethyl esters of 8′‐apo‐β‐carotenoic acid or canthaxanthin, with oils or vegetable fats, starch, gelatin, sucrose or dextrin, suitable for coloring foods) and NCM 32041919 (other preparations based on carotenoids).

**TABLE 1:** Brazilian import and export of carotenoids and their derivatives from 2014 to 2024.

	NCM 32041810			NCM 32041911			NCM 32041912			NCM 32041919		
	Exports ($)	Imports ($)	Net weight E + I (kg)	Exports ($)	Imports ($)	Net weight E + I (kg)	Exports ($)	Imports ($)	Net weight E + I (kg)	Exports ($)	Imports ($)	Net weight E + I (kg)
2014	0	0	0	83 965.00	1 955 531.00	130 551	40 720.00	2 844 689.00	75 785	1 850.00	2 386 717.00	1060 222
2015	0	0	0	245 502.00	1 891 107.00	89 350	20 538.00	3 654 388.00	99 733	2 666.00	606 993.00	243 952
2016	0	0	0	70 078.00	4 915 469.00	130 908	240.00	3 947 536.00	122 936	521.00	1 575 312.00	523 062
2017	0	0	0	50 591.00	3 647 900.00	76 701	53 402.00	4 201 783.00	136 082	1 248.00	1 416 460.00	450 900
2018	0	0	0	88 782.00	3 480 568.00	72 988	124 067.00	5 602 962.00	147 316	6 826.00	1 422 358.00	363 089
2019	0	0	0	409.00	3 463 796.00	83 591	97 077.00	4 099 618.00	114 256	8747.00	990 038.00	291 207
2020	0	0	0	34 305.00	3 621 373.00	89 233	12 562.00	5 725 106.00	153 306	9 579.00	806 280.00	145 678
2021	0	0	0	37 336.00	8 589 715.00	159 366	72 574.00	5 932 385.00	152 108	41 664.00	989 606.00	262 798
2022	133 990.00	4 462 667.00	104 499	21.00	1 821 837.00	36 076	625.00	2 593 828.00	77 607	2884.00	319 951.00	84 038
2023	29 137.00	9 553 643.00	194 016	0	0	0	0	0	0	0	0	0
2024	187 915.00	7 397 027.00	159 279	0	0	0	0	0	0	0	0	0

*Note*: Brazilian foreign trade statistics data portal (https://comexstat.mdic.gov.br/en/geral/112491) NCM 32041810 and NCM 32041911 = carotenoids. NCM 320419912 = preparations containing β‐carotene, methyl, or ethyl esters of 8′‐apo‐β‐carotenoic acid or canthaxanthin, with oils or vegetable fats, starch, gelatin, sucrose, or dextrin, suitable for coloring foods. NCM 32041919 = other preparations based on caratenoids. E + I = exports + imports. Period from January 2014 to September 2024.

In Brazil, according to the statistical data from the Brazilian Foreign Trade [72], $1 459 821.000 were exported, representing about 24 602 kg of carotenoids and their derivatives in the period between January 2014 and September 2024 (Table 2), while $103 916 643.00 was spent on imports with 5 806 031 kg in the same period.

**TABLE 2:** Overview of Brazilian import and export of carotenoids and their derivatives from 2014 to 2024.

	Subtotal				
Year	Exports ($)	Imports ($)	Net weight E + I (kg)		
2014	126 535.00	7 186 937.00	1 266 558		
2015	268 706.00	6 152 488.00	433 035		
2016	70 839.00	10 438 317.00	776 906		
2017	105 241.00	9 266 143.00	663 683		
2018	219 675.00	10 505 888.00	583 393		
2019	106 233.00	8 553 452.00	489 054		
2020	56 446.00	10 152 759.00	388 217		
2021	151 574.00	15 511 706.00	574 272		
2022	137 520.00	9 198 283.00	302 220	**Subtotal**	
2023	29 137.00	9 553 643.00	194 016	**Net weight detached**		
2024	187 915.00	7 397 027.00	159 279	**Net weight E (kg)**	**Net weight I (kg)**
Total	1 459 821.00	103 916 643.00	5 830 633	24 602	5 806 031

*Note*: Brazilian foreign trade statistics data portal (https://comexstat.mdic.gov.br/en/geral/112491). Period from January 2014 to September 2024.

**FIGURE 4:**
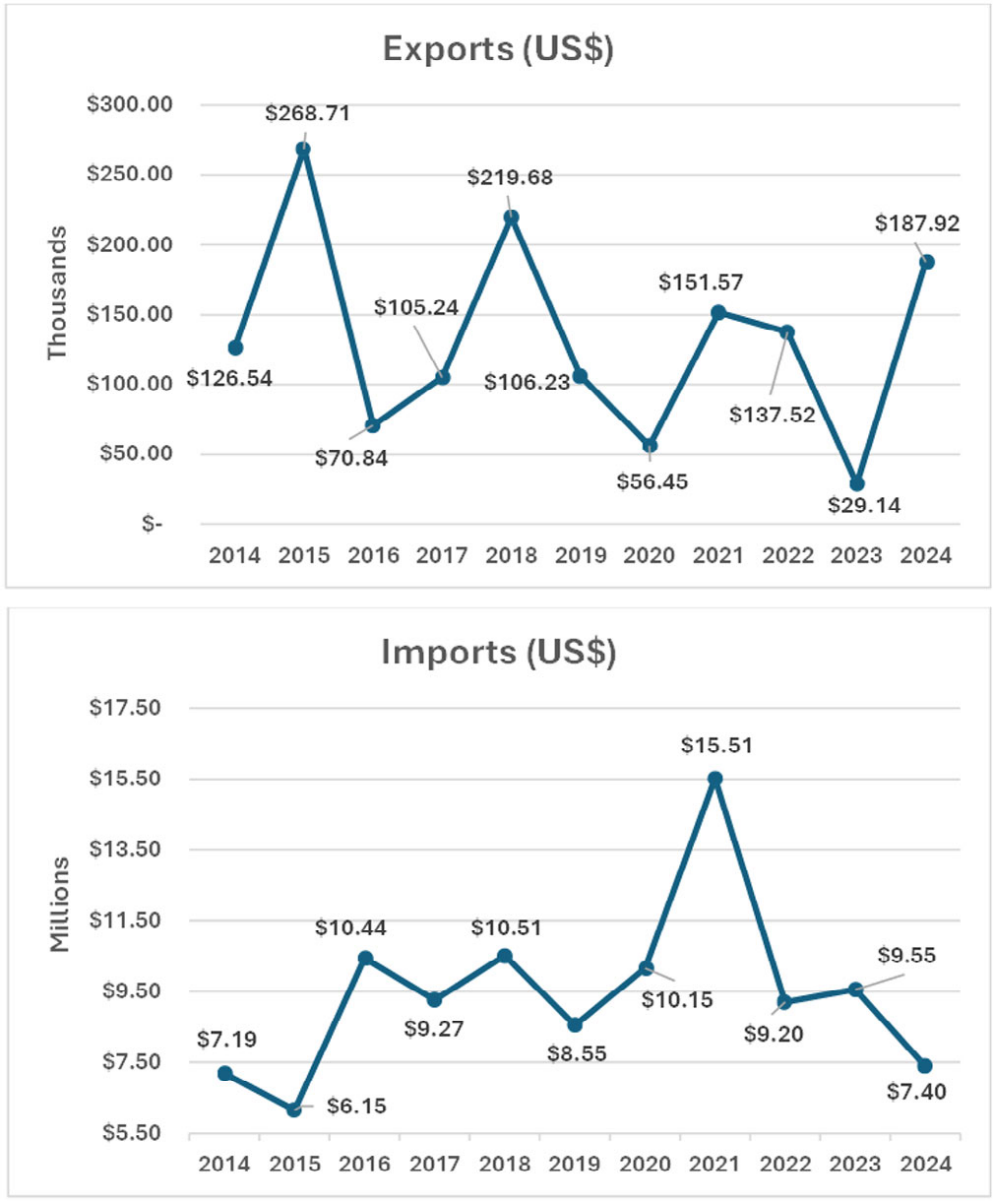
Illustration of Brazilian export (in thousand per year) and import (in million per year) of carotenoid and their derivatives.

Considering that Brazil hosts a huge biodiversity of plants and microorganisms that are carotenoid producers, and based on these data, it becomes clear that Brazil imports a large quantity of carotenoids for industrial applications, by spending about $103 million and exports only around 1.5 million (Figure 4), probably due to a lack of initiatives and investments in the industrial sector to develop it. We hypothesize a positive scenario to strengthen the Brazilian carotenoid market considering alternatives from our biodiversity to produce carotenoids, resulting in increased exports and decreased imports. To achieve this goal, systematic scientific research must be stimulated with technological and biotechnological approaches to undercover new plant or microbial sources, as well as sustainable processes to produce high yields of carotenoids.

## 6 Yeasts of Genus *Rhodotorula* Found in the Brazilian Biomes

Brazil is one of the countries with one of the richest biodiversity in the world, holding six biomes: Amazon, Cerrado, Caatinga, Atlantic Forest, Pantanal, and Pampa [73].

The Amazon is the largest area of remaining tropical forest in the world (3.5 million km^2^) and one of the most important ecosystems in the world. The Amazon biome holds one of the largest existing biodiversity, which is vital for the functioning of the biosphere. This biome can provide many goods and services for humanity with considerable economic and social value, especially for food and pharmaceutical industries, among others [74, 75].

Cerrado (Brazilian savanna), is predominant in the central region of Brazil, present in the states of Mato Grosso, Mato Grosso do Sul, Tocantins, Goiás, western Minas Gerais, and Bahia, and in the Northeast. It also occupies some parts of Pará, Maranhão, Rondônia, São Paulo, and Paraná [76]. The national area is 2 million km^2^, representing 23% of the Brazilian territory and 11% of South America. Its vegetation has species capable of withstanding extreme environments [76, 77, 78].

Caatinga, a biome 100% hosted by Brazil, is a semiarid ecosystem that corresponds to 10% of the Brazilian territory and 60% of the northeast region. Its environmental and chemical characteristics are unique [79, 80]. It has dry and thorny shrub vegetation, and due to its temperature and little rainfall it is also categorized as a seasonal tropical forest [80, 81].

The Atlantic Forest is one of the most fragmented tropical/subtropical forests in the world, located along the entire Brazilian coast [82]. Its vegetation is seasonal semi‐deciduous; the climate has two well‐defined seasons: cold dry and hot rainy. The average temperature is 19°C with rainfall with an average volume of 13 nm [83, 84].

Pantanal, the smallest biome in Brazil, is one of the wettest regions in the world, and covers 140 000 km^2^ in Brazil. This biome is predominant in the states of Mato Grasso and Mato Grosso do Sul [85, 86, 87]. This large alluvial plain represents a complex of interconnected aquatic, terrestrial and wetland ecosystems. The dry and wet seasons are well defined, with rainfall in the summer [85, 88].

Pampa is the second smallest biome in the country, occupying an area of 176 496 km^2^. It is present in the state of Rio Grande do Sul [89, 90]. The region is a temperate zone, with well‐defined seasons, a rainy subtropical climate, without dry periods and negative temperatures in winter [91]. This biome is not very dense, and its vegetation consists of herbaceous plants and shrubs [89, 90].

Concerning biodiversity, all the Brazilian biomes can be seen as specific incredible habitats for a substantial number of animals, plants, including macroscopic and microscopic beings. Regarding microorganisms, it will be focus on yeasts of the genus *Rhodotorula* present in all the Brazilian biomes to summarize where they were investigated, which species inhabit these sites, and the relevant gaps in relation to what other literatures has not yet addressed (Table 3).

**TABLE 3:** Yeasts of genus *Rhodotorula* found in the Brazilian biomes as promising natural sources of carotenoids.

Brazilian biome	Yeast	Origin	Collection site	Cultivation medium	Total carotenoid contents	Carotenoid composition	Carotenoid identification	Yeast identification	Biomass (g/L)	Reference
Amazon	*R. mucilaginosa*	Bosque da Ciência—(INPA), Manaus, AM	*Garcinia macrophylla* Mart. leaf litter; *Theobroma cacao* L. fruits	Synthetic medium (YEPD)	601.0 µg/g	NR	Thin‐layer chromatography (TLC)	PCR, de novo sequencing, BLAST	6.7	[20]
Amazon	*R. mucilaginosa, R. glutinis*, *R. aurantiaca*, and *R. minuta*	Maracá Island/RR	Soil from the Maracá Ecological Station	Synthetic medium (YM)	NR	NR	NR	PCR, de novo sequencing, BLAST	NR	[18]
Amazon	*R. mucilaginosa*	Sossego mine—Canaã dos Carajás/PA	Water in copper waste lake	Synthetic medium (YEPD)	NR	NR	NR	Microscopy	NR	[92]
Amazon	*Rhodotorula* sp.	Villages of Ipiranga (second Special Border Platoon—PEF), Vila Bitten court (third PEF) and Cucuí—AM	Rivers in the localities and close to the tribes	Synthetic medium (SDA)	NR	NR	NR	Microscopy	NR	[19]
Amazon	*Rhodotorula* sp.	Belém/PA	Brazil nut	Synthetic medium (nutrient agar)	NR	NR	NR	MIDI microbial identification system	NR	[16]
Cerrado	*R. glutinis* *R. mucilaginosa* *Rhodotorula graminis* *R. aurantiaca* *R.lactosa*	Barra dos Garças; Pontal do Araguaia and Aragarças—GO; UNESP—São José do Rio Preto—SP	Sheets; fruits; flower; tree bark; Earth; insects	Synthetic medium (YM)	118.84 µg/g 790.31 µg/L	NR	NR	PCR	6.65	[97]
Cerrado	*R. toruloides and Rhodotorula* sp.	GO‐194—Aragarças/GO (latitude −15.926710, longitude −52.234504)	Soil, leaves, and flowers	Synthetic medium (YM)	461.59–1349.54 µg/L	NR	NR	PCR	5.36–12.83	[12]
Cerrado	*R. mucilaginosa*	Bacaba/MT; Lagoa/PA; Ponte Alta/PA	Unspecified vegetation	Synthetic medium (YM)	NR	NR	NR	PCR, and comparison with GenBank (BLAST, NCBI)	NR	[17]
Cerrado	*R. mucilaginosa*	Rocky fields Serra do Cipó National Park/MG; south of Serra do Espinhaço/MG	Water samples from bromeliads (*Papiliotrema laurentii phytotelmata*)	Synthetic medium (YM)	NR	NR	NR	PCR	NR	[98]
Cerrado	*R. mucilaginosa*	Barra do Bugres/MT	Fruits	Synthetic medium (YPD)	NR	NR	NR	NR	NR	[99]
Cerrado	*R. mucilaginosa* and *R. diobovata*	Arinos/MG	fruits of *Annona crassiflora* Mart. (araticum), *Syagrus oleracea* Becc (guariroba coconut), *Butia capitata* (azedo coconut)	Synthetic medium (YM)	NR	NR	NR	Sanger method skin sequencing	NR	[13]
Cerrado	*R. lactosa*	Barra do Garças/MT	Ground; sheets; tree bark; flower petals	Synthetic medium (YM)	194.17 µg/g to 776.67 µg/L || CCD = 131.55–147.36 µg/g and 1019.84–1387.63 µg/L	NR	NR	PCR	6.60 || CCD = 5.05–10.72	[100]
Caatinga	*R. mucilaginosa* (former *R. rubra*)	Not specified	Oral cavity of the snake *Lygophis dilepis* and the skin of the frog *Rhinella jimi*	Synthetic medium (YEPD)	NR	NR	NR	PCR	NR	[101]
Caatinga	*R. acheniorum*, *R. aurantiaca*, *R. glutinis* *R. mucilaginosa* *R. aurantiaca*	Garanhuns, São Vicente Férrer and Macaparana/PE	*Vitis labrusca* in Garanhuns/PE; *Coffea arabica* leaves in Garanhuns/PE; São Vicente Férrer and Macaparana	Synthetic medium (PDA; SDA; YPG)	NR	NR	NR	Morphological identification by microscopy	NR	[102]
Caatinga	*R. glutinis* *R. mucilaginosa*	Pici Campus, UFC—Fortaleza/CE	Ground	Synthetic medium (MGYP, potato‐glucose agar)	NR	NR	NR	Spectrophotometry with VITEC2 mass spectrum from BioMéreiux SA	NR	[103]
Caatinga	*Rhodotorula* sp.	Furna do Morcego cave—Catimbau National Park (PARNA Catimbau) in Pernambuco	Carven air, guano, bat oral cavity, wings, and hair	Synthetic medium (SDA)	NR	NR	NR	Morphological (microscopy), PCR and sequencing	NR	[104]
Caatinga	*R. glutinis*	Caatinga in the state of PE	Semiarid soil	Synthetic medium (Sabouraud) + supplementation with crude glycerin and corn liquor	123.12–156.65 µg/g	β‐Carotene	HPLC	NR	4.98	[105]
Caatinga	*R. mucilaginosa*, *R. glutinis Rhodotorula* sp.	Morro da Pioneira—Serra da Jiboia region—BA	Bromeliad flowers and water	Synthetic medium (YE)	NR	NR	NR	API 20 C AUX System	NR	[106]
Atlantic Forest	*R. glutinis*	Campinas and Holambra/SP	Tomato puree, soil from Campinas, sugar cane leaves and soil from Holambra	Synthetic medium (GPYM)	54.7–61.4 µg/g	β‐Carotene and torulene	HPLC	Comparative analysis of morphology, physiological and metabolic characteristics	6.7–8.7	[107]
Atlantic Forest	*R. mucilaginosa*	São José do Rio Preto/SP	Soil, leaves, flowers, tree bark, and grass	Synthetic medium (YM)	111.69 µg/g 283.35 µg/L	NR	NR	NR	3.27–6.30	[108]
Atlantic Forest	*R. mucilaginosa*	Bairro Novo and Casa Caiada beaches in Olinda/PE	Sand and sea water	Synthetic medium (SDA and YE)	NR	NR	NR	Physiological observation by microscopy	NR	[11]
Atlantic Forest	*Rhodotorula* sp.	Southeastern Brazil	Not specified	Synthetic medium (GYMP)	NR	NR	NR	Not specified	NR	[109]
Atlantic Forest	*R. mucilaginosa*	Catanduva, Colina, Elisiário, and Novais/SP	Orange leaves (*Citrus sinensis*)	Synthetic medium (YM)	NR	NR	NR	ITS sequencing sequence from BLAST analysis and scanning electron microscopy	NR	[110]
Atlantic Forest	*R. paludigena*	Ilhéus/BA	Cocoa tree (leaves, flowers, and fruits)	Synthetic medium (YEPD)	NR	NR	NR	Common microscopy and PCR and comparison with NCBI	NR	[111]
Atlantic Forest and Pampa	*R. mucilaginosa*	Paraná, Santa Catarina and Rio Grande do Sul	Freshly extracted apple must	Synthetic medium (YALYS, Rose Bengal chloramphenicol, YMA and GPYB)	NR	NR	NR	Satellite prepared Micro/mini‐PCR (MPP‐PCR) fingerprint method.	NR	[112]
Pampa	*R. mucilaginosa*	Sul‐Riograndense Shield and the East Coast of the South region	Soil, bark, flowers leaves and fruits	Synthetic medium (YM)	230–360 µg/L	β‐Carotene and β‐cryptoxanthin	HPLC	Mini/microsatellite‐primed PCR technique (MSP‐PCR)	12.02–12.49	[113]
Pampa	*R. mucilaginosa*	Middle Coast and South Shield‐Rio‐Grandense/RS	Not specified	Synthetic medium (YM)	17.6–83.5 µg/L	Astaxanthin, Lutein and β‐carotene	HPLC	Mini/microsatellite‐primed PCR technique (MSP‐PCR)	6.4	[10]
Pampa	*R. mucilaginosa*	Rio Grande do Sul	Aerial parts of plants	Synthetic medium (DAS)	NR	NR	NR	Microscopy	NR	[95]
Pantanal	*R. aurantiaca*; *R. glutinis*; *R. minuta*; *R. mucilaginosa*	Cuiabá and Várzea Grande/MT	Dust of libraries	Synthetic medium (Sabourand agar)	NR	NR	NR	NR	NR	[114]

Abbreviations: AM, Amazonas State; BA, Bahia State; CCD, central composite design; CE, Ceará State; GO, Goiás State; GPYM, glucose, peptone, yeast extract, malt; GYMP, glucose, yeast extract, malt, peptone; HPLC, high‐performance liquid chromatography; INPA, Instituto Nacional de Pesquisas da Amazonia; MG, Minas Gerais State; MGYP, malt extract glucose yeast extract peptone agar; MT, Mato Grosso State; NCBI, National Center for Biotechnology Information; NR, not reported; PA, Pará State; PCR, polymerase chain reaction method; PDA, potato dextrose agar; PE, Pernambuco State; RR, Roraima State; RS, Rio Grande do Sul State; SDA, Sabouraud dextrose agar; SP, São Paulo State; UFC, Federal University of Ceará; YE, yeast extract; YEPD, yeast extract peptone dextrose; YM, yeast malt; YMA, yeast mannitol agar; YPD, yeast peptone dextrose; YPG, yeast extract peptone glycerol.

In the Amazon region, some studies reported the presence of *R. mucilaginosa* in samples from mines in Southeast Pará using its dead mass for nanobioremediation, recovery of silver metals and absorption of metallic copper ions in wastewater [92, 93]. Furthermore, species were found in soils at the Maracá Ecological Station in Roraima, with emphasis on *R. mucilaginosa*, *R. glutinis*, and *R. minuta*. In this study, resistance to mycocins in *Williopsis saturnus*, *Issatchenkia* sp., and *Saccharomyces exiguus* also isolated from the same region [18], and *R*. *mucilaginosa* was isolated in samples of non‐keratinophilic soil [94]. In a recent study, strains of *R. mucilaginosa* isolated from *Garcinia macrophylla* Mart. leaf litter and *Theobroma cacao* L. fruits demonstrated the total carotenoid production of 601.0 µg/g (RGM42), 362 µg/g (RTC42), and 351 µg/g (RTC45), and biomass of 6.7, 6.8, and 6.42 g/L, respectively [20]. During the data collection, there were few studies on yeasts of the *Rhodotorula* genus in Amazonia compared to other biomes, which is surprising since this biome is a biodiversity hotspot as it concentrates endemic species of plants, invertebrates, and yeasts [95, 96]. The reasons may be diverse, from lack of interest or lack of investment in research that discovers the presence of this genus in the Amazon region to the difficulty in going to places to collect samples.

In studies with the objectives of describing the occurrence and diversity of yeasts cultivable in Cerrado fruits and evaluating the potential of isolated strains to produce carotenoids, some species of *Rhodotorula* were found (*R. glutinis*, *R. mucilaginosa*, *R. graminis*, *R. aurantiaca*, *R. lactosa*, *Rhodotorula diobovata*, *R. toruloides*). These species were present in leaf samples, fruits (*Anacardium humile* Mart., *Annona crassiflora* Mart., *Butia capitata* Mart., *Caryocar brasiliense* Cambess, *Eugenia dysenterica* DC., *Hancornia* *speciosa* Gomes, *Hymenaea stigonocarpa* Mart. Ex Hayne, *Mauritia flexuosa* Lf., *Passiflora cincinnata* Mast., *Psidium cattleyanum* Sabine, *Solanum lycocarpum* A.St‐Hil, *Syagrus oleracea* Becc and *Talisia esculenta* Radlk), flowers, tree bark, soil, and insects, although plant and insect species were not mentioned in the studies [12, 78, 97].

In the research of Machado and Bianchi [100], *Rhodotorula* yeasts were isolated from soil and cultivated in synthetic/commercial medium (yeast malt [YM]), followed by identification by DNA analysis (PCR), and the authors highlighted *R. lactosa* with high carotenoid production of 194.17 µg/g (dry basis) and 776.67 µg/L (wet basis) in 6.6 g/L of biomass. After applying the optimization of carotenoids production by the central composite design (DCC) 22, with low agitation (130 rpm) and at room temperature (25°C), the production was favored, reaching 188.40 µg/g of carotenoids in 5.05 g/L of biomass. The same authors showed an expressive increase in the volumetric production of carotenoids by reducing the components of the culture medium associated with elevated levels of glucose and concluded that *R. lactosa* needs tiny amounts of nitrogen sources and substantial amounts of carbon sources.

In an investigation of Machado [97], the authors selected wild carotenogenic yeasts from the Cerrado biome, specifically from soils, flowers, leaves, and fruits, but without specifying the species of flowers, leaves and fruits, and not even showed on map the exact location of yeast‐soil isolated, which demonstrated some lack of information. The medium used for the collection was YM, being potato dextrose agar (PDA) used for the characterization of isolated and pure strains, glucose, yeast extract, malt extract and monobasic sodium phosphate (GYMP) for the maintenance of strains, and finally YM for the inoculum.

In the Caatinga biome, studies demonstrated the presence of *Rhodotorula* in samples taken from the oral cavity and cloaca of reptiles, from the surface of the skin of amphibians [101], bats (from the oral cavity, wings, and hair) and in caves (from the air and guano—natural organic fertilizer which originates from the mixture between bat excrement and cave minerals), but this research did not focus on the production of carotenoids [104]. Studies by Andrade et al. [105] and Mendes‐Silva et al. [115] reported the presence of β‐carotene produced by *R. glutinis* in semiarid soil samples from the Caatinga biome. According to the authors, the total carotenoid content was 123.12 µg/g, in the absence of light. With the supplementation of crude glycerin, steep corn liquor and peptone, there was an increase in production, reaching 127.86 µg/g after 96 h of cultivation, under the influence of blue LED light. During the optimization runs, the presence of β‐carotene was confirmed by HPLC in the supplemented media plus the addition of blue LED and its exclusion, with the yield being higher in the absence of light (100.60 µg/g) than in its presence (82.02 µg/g).

On the other hand, several studies were developed in the Atlantic Forest region, in the state of São Paulo [67, 109, 116]. A greater variety of yeast species were found: *R. mucilaginosa*, *R. graminis*, with 8.7 g/L of biomass production and 61.4 µg/g of carotenoid production [107], being *R. glutinis* [106] and *R. minuta* [117] also found in this region. These *Rhodotorula* yeasts were isolated from samples of soil, leaves, fruits, flowers, and processed foods, with β‐carotene predominant in *R. graminis*, and torulene the predominant carotenoid in *R. mucilaginosa* [107]. Moreover, *Rhodotorula* yeasts were also isolated from fig tree species (*Ficus cestrifolia*, *F. lushnathiana*, and *Coussapoa microcarpa*) [117], from flowers belonging to the species *Sobralia liliastrum*, *Epidendrum secundum*, *Fabaceae* sp., *Mirtaceae* sp., *Lamiaceae* sp., and from water from a pond bromeliad belonging to the species *Alcantarea nahoumii* [106].

In the Pampa biome, the presence of *Rhodotorula* in the Rio Grande do Sul State, on the east coast of the southern region, mainly represented by *R. mucilaginosa*, was found in samples of aerial parts of plants, soil, bark of trees, leaves, fruits, and apple must, but the environmental species from which these samples were taken were not informed. In this species, β‐carotene, β‐cryptoxanthin, astaxanthin, and lutein were the carotenoids identified [10, 95, 113], being 13.5 g/L of biomass obtained in YM medium after 96 h. From this biomass, 93.9 µg carotenoids/g of were observed and 1068.5 µg carotenoids/L were obtained, with high productivity in the YM medium (83.5 µg carotenoids/L/h) [10]. The results obtained by Otero et al. [113], also in YM medium, showed biomass of 11.23 g/L and 280 µg carotenoids/L.

Regarding the Pantanal biome, during the information gathering, few studies have found that reported *Rhodotorula*. In these studies, samples of fruits, flowers and dusts were collected; and some species identified as *R. aurantiaca*, *R. glutinis*, *R. minuta*, and *R. mucilaginosa* [114, 118, 119]. Furthermore, the exact locations (city, municipality, environmental park, or other location) from where the *Rhodotorula* yeasts had taken were not reported, nor the carotenoid determination were conducted in most studies.

**FIGURE 5:**
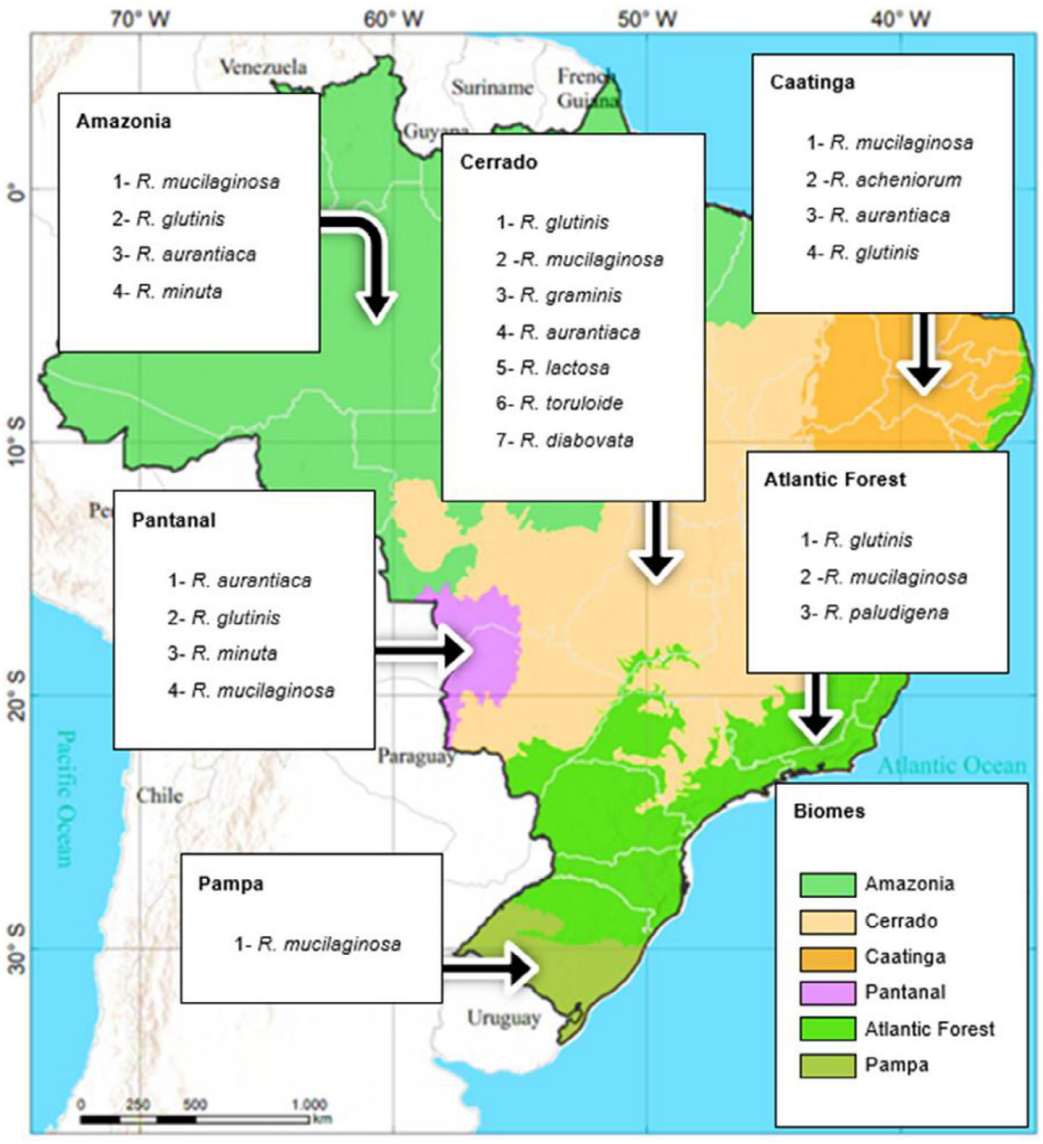
Profile of *Rhodotorula* species in Brazilian biomes. Adapted from Instituto Brasileiro de Geografia e Estatística (2024).

As demonstrated in Table 3 and Figure 5, the Cerrado biome exhibited the highest number of *Rhodotorula* yeast species, followed by the Caatinga, Pantanal, and Amazon biomes. However, the Cerrado exhibited the highest concentrations of carotenoid‐producing strains with the species *R. toruloides* and *Rhodotorula* sp. (639.05 and 609.80 µg/L, respectively), closely followed by the Amazon (601.0 µg/g—RGM42, 362 µg/g—RTC42, and 351 µg/g—RTC45). Conversely, there is a paucity of reports on yeasts in the Atlantic Forest and Pampa biomes, particularly in the Pampa. This finding suggests a lacuna in the field of bioprospecting research in these regions and a potential diminution of novel discoveries concerning the microbial biodiversity of these environments. Moreover, the existing studies have not investigated the use of technologies such as HPLC, for example, for appropriate identification of carotenoids, as evidenced by the NR designation in Table 3, showing such shallow approach of some previous studies.

## 7 Optimization of Carotenoid Production in *Rhodotorula* Strains From Brazilian Biomes

In general, optimization procedures varying the process conditions of biotechnological cultivation were the most frequent strategies adopted by the researchers aiming to increase carotenoid production. The carotenoid contents are commonly reported as total contents, by spectrophotometry, which does not allow the precise assignment of the carotenoid composition, the results expressed as equivalents of β‐carotene based on the specific absorption coefficient of β‐carotene in a selected analytical solvent. However, one of the main gaps observed in our review is the lack of information regarding the individual composition of carotenoids produced by the isolated *Rhodotorula* yeasts, at least, by chromatographic techniques. By this review, only four papers confirmed the carotenoid composition by high performance liquid chromatography (Table 3). In all the Brazilian biomes, the major identified carotenoid was β‐carotene, which can be seen as an excellent observation, since it is the most used carotenoid by the food, pharmaceutical, and cosmetic industries [2, 120].

According to the selected papers [97, 100], the production of carotenoids by *Rhodotorula* yeasts seems to be high in soil and flowers, varying from 50–194 µg/g. The *R. lactosa* (QWRR) sample was taken from Cerrado soil, which showed an orange color, which was identified by PCR as *R. lactosa*, with the highest carotenoid contents (194.17 µg/g) and biomass of 4.0 g/L. The second highest carotenoid production (118.84 µg/g and biomass 6.65 g/L) was reported for *R. glutinis* (ADSF) from flower samples, whose strain showed a pinkish color.

After maximizing the biotechnological procedure by varying stirring and temperature of cultivation, high contents of carotenoids were observed for *R. lactosa* (188.4 µg/g in 5.05 g/L of biomass) and for *R. glutinis* (92.52 µg/g in 8.14 g/L of biomass). For both the yeasts cultivation, changes in the total contents of carotenoids and biomasses seemed to occur at the lowest agitation level (130 rpm) and temperature (25°C) after 144 h of cultivation at initial pH of 5 [97]. Furthermore, in this same study, *R. mucilaginosa*, which was obtained at the São Paulo State University (UNESP, São José do Rio Preto, SP, Brazil), belonging to both the Cerrado and Atlantic Forest biomes, with no specification about the collection site, produced 252.99 µg carotenoids/g in 16.46 g/L of biomass, cultivated at 25°C, pH of 6.5 and agitation at 130 rpm.

In another study also by [12], with samples of soil, leaves, and fruits, and again without specifying the plant species, the microorganisms were inoculated in YM medium, which later showed a pinkish color. After identification by PCR, it was found that the colonies were *R. toruloides* and *Rhodotorula* sp., and they showed carotenoid production of 639.05 and 609.80 µg/L, respectively. After the optimization procedure, the authors concluded that *R. toruloides* required minimal amounts of nitrogen to maintain cell development and large amounts of carbon for carotene synthesis, in addition to pH 6 for the production of carotenoids (106.92 µg/g).

The species *R. mucilaginosa* was found in the Cerrado region in the states of Mato Grosso (municipality of Bacaba), Pará (in Lagoa and Alta) [17], Minas Gerais (Parque Nacional da Serra do Cipó and Serra do Espinhaço) [98], and in another city in the Mato Grosso state, called Arinos [13].

Comparing the gathered information, it is clear that some investigations did not specify relevant information such as the plant and fruit species [95, 113] in the Pampa biome, which could make it difficult to reproduce the study. The same situation occurred in studies in the Pantanal biome, with the lack of the exact location for sample collection [114, 118, 119]. In the Atlantic Forest, plant species used were not mentioned [107, 109]. Most research conducted in this biome used samples from plant sources, indicating a wide presence of *Rhodotorula* in them. However, few studies were noted with qualitative and quantitative results on carotenoid production.

*Rhodotorula* yeasts are often found in the Caatinga and Cerrado in soil samples, but also quite often in plant samples [12, 97, 105, 108, 115]. Furthermore, in the Cerrado biome, studies showed a greater variety of carotenogenic *Rhodotorula* species in relation to the other biomes, namely: *R. glutinis*, *R. graminis*, *R. aurantiaca*, *R. lactosa*, and *R. mucilaginosa* [97].

As observed by Igreja et al. [2], the yeast species belonging to the *Rhodotorula* genus are affected by factors such as pH, temperature, and agitation. The pH range of the samples exhibited a variation from 4 to 6.2, the temperature ranged from 15 °C to 34 °C, and the agitation speed ranged from 100 to 180 rpm. The results of the study indicate that pH has a significant impact on carotenogenesis in yeast, exhibiting a preference for more acidic pH values. However, low pH values have been observed to result in yeast growth inhibition and a reduction in carotenoid production. Regarding temperature, low values generally do not promote cell growth and carotenoid production. Conversely, elevated levels have been observed to denature enzymes involved in carotenogenesis and to impede cell growth. The aeration and agitation parameters have been demonstrated to enhance carotenoid production capacity, though this effect is only observed in conjunction with pH and temperature. Furthermore, factors such as light irradiation and substrate composition have been demonstrated to affect the carotenoid yield. The carotenoid biosynthesis of some yeasts can be influenced by light exposure due to a response mechanism to that exposition. In addition, sources rich in C (carbon) and N (nitrogen) have been shown to boost carotenoid production in yeasts. As demonstrated herein, yeasts from the *Rhodotorula* genus exhibit a remarkable capacity for adaptation, which elucidates their presence across all Brazilian biomes.

## 8 Principal Samples, Separation Methodologies and Biotechnology Approaches

In addition to optimization by varying cultivation conditions, another approach used to increase production and yield is molecular biology techniques such as CRISPR‐Cas9 [121], CRISPR‐Cas12a [122], CAPTURE (Cas‐12 Assisted Precise Target cloning Using in vivo Cre‐lox REcombination) [123], TAR (Transformation‐Associated Recombination) [124, 125] which consist of gene editing and cloning of genes of interest, in this case, genes that encode carotenoids

Most studies have reported soil, tree leaves and bark, flowers and fruit as the main types of samples where yeasts can be found [12, 18, 97]. The most commonly used forms of collection are the removal of material from the environment by swabbing with sterile swabs and water and peptone on the surfaces of leaves, flowers, fruit and tree bark, removal of soil samples with garden shovels and sterilized or sanitized drills and the use of sterilized plastic bags, with final manipulation in the laboratory [100, 103, 126].

Given their fat‐soluble nature, the proper separation of carotenoids is essential for both research and industrial applications. The methodologies employed in this study generally combine extraction and chromatographic techniques. Among the most applied techniques are High Performance Chromatography (HPLC) [C18 column; coupling with UV‐Vis, PDA (diode‐array), and FLD (fluorescence detector); mobile phases: acetonitrile, methanol, and petroleum ether or ethyl acetate]. Thin‐layer chromatography (TLC) is a technique that is most often employed in preliminary analyses or screenings. It utilizes organic solvents and allows for visual detection under white light or UV light. The use of densitometry is also a common practice. Gas chromatography is a less common method for analyzing intact carotenoids; however, it is a useful technique for the analysis of oxidized carotenoids or degradation products following derivatization. The derivatization process is necessary because carotenoids are thermolabile, meaning they are unstable at high temperatures. To analyze carotenoids, they must be converted into volatile forms. The following detectors are used: The utilization of FID (flame ionization) and MS (mass spectrometry) in conjunction with each other is imperative for the study of metabolites and oxidized carotenoids. The extraction process, which utilizes organic solvents, is a prerequisite to chromatographic separation. The solvents employed in this process include acetone, ethanol, hexane, and chloroform. These solvents are often utilized in conjunction with liquid‐liquid extraction or partitioning with aqueous saline solutions or buffers. This approach is employed to extract both the carotenes and the xanthophylls present in the samples. Assisted extraction of carotenoids is an optional procedure that is important for yield and selectivity. These include ultrasound (UAE), microwave (MAE), and supercritical (SFE, with supercritical CO_2_). Assisted extraction is the most common method employed for the isolation of carotenoids from plant matrices. This extraction technique exhibits high selectivity and does not necessitate the use of toxic organic solvents [127, 128, 129, 130, 131].

In summary, based on this review and the Figure 5, *R. mucilaginosa* was found in all the Brazilian biomes, seemed to be the most frequent *Rhodotorula* in Brazil. The Cerrado biome had the largest number of *Rhodotorula* species, seven in total (*R. glutinis*, *R. mucilaginosa*, *R. graminis*, *R. aurantiaca*, *R. lactosa*, *R. toruloide*, and *R. diabovata*), followed by Caatinga with five (*R. mucilaginosa*, *R. acheniorum*, *R. aurantiaca*, *R. glutinis*, and *R. aurantiaca*), Amazon with four (*R. mucilaginosa*, *R. glutinis*, *R. aurantiaca*, and *R. minuta*) and Pantanal with also four (*R. aurantiaca*, *R. glutinis*, *R. minuta*, and *R. mucilaginosa*).

## 9 Conclusion and Future Perspectives

Carotenoids are greatly beneficial compounds with high biological and technological properties to be used as natural food colorants and antioxidants. These properties have been stimulating the global market of carotenoids for industrial application as relevant opportunities to increase exportation in Brazil. However, current consumers demand natural sources of food additives to accomplish the adoption of healthier habits, but mostly commercially available carotenoids are still produced by chemical synthesis as such method results in high yields and less industrial cost.

*Rhodotorula mucilaginosa* is the predominant yeast species in all Brazilian biomes. As demonstrated in the state of Pará, the object of study has applications in the bioremediation of heavy metals. The species exhibited optimal carotenoid yields, with concentrations of 601.0 µg/g, 362 µg/g, and 351 µg/g recorded in the Amazonas region. As demonstrated in this research, the combination of factors, including temperature, agitation, aeration, exposure to light, pH of the growing medium, and the presence of residues in the cultures, has the capacity to affect the yield of carotenoids and the production of biomass in the ten species shown herein. This evidence suggests that species of the *Rhodotorula* genus possess adaptive potential and can be subjected to improvements in their yields, as well as in the use of waste from industries.

Our review intended to highlight alternative biotechnological options to produce carotenoids by *Rhodotorula* yeasts that do not depend on availability, seasonality or other agronomic and geographic factors as compared to plant sources.

All the Brazilian biomes have high potential to contribute with a number of *Rhodotorula* yeasts to be further investigated to the production of selected carotenoids to stimulate the growing of national bioeconomy. It becomes clear that all the Brazilian biomes need more research focused on the identification of carotenoids sources as very few have been developed, and much more can still be discovered and improved in this area.

## Author Contributions

Conceptualization: David Lucas and Renan Chisté. Methodology: David Lucas and Renan Chisté. Investigation: David Lucas and Renan Chisté. Resources: Renan Chisté. Data curation: David Lucas. Writing – original draft: David Lucas and Renan Chisté. Writing – review and editing: David Lucas and Renan Chisté. Visualization: David Lucas and Renan Chisté. Supervision: Renan Chisté. Project administration: Renan Chisté. Funding acquisition: Renan Chisté. All authors have read and agreed to the published version of the manuscript.

## Ethics Statement

The authors have nothing to report.

## Conflicts of Interest

The authors declare no conflicts of interests.

## Data Availability

All data related to this manuscript are available in the form of Figures and Tables in the manuscript.
